# Expression Analysis of *FGF/FGFR* and *FOX* Family Proteins in Mucosal Tissue Obtained from Orofacial Cleft-Affected Children

**DOI:** 10.3390/biology10050423

**Published:** 2021-05-10

**Authors:** Māra Pilmane, Nityanand Jain, Zane Vitenberga-Verza

**Affiliations:** Department of Morphology, Institute of Anatomy and Anthropology, Riga Stradinš University, LV-1007 Riga, Latvia; mara.pilmane@rsu.lv (M.P.); zane.vitenberga-verza@rsu.lv (Z.V.-V.)

**Keywords:** cleft palate, cleft lip, immunohistochemistry, in-situ hybridization, inflammation, FGF/FGFR, FOXE1, FOXO1

## Abstract

**Simple Summary:**

Craniofacial development is an intricate and delicate process in normal embryogenesis requiring spatiotemporal release of various mediators/proteins that provide communication between different cell/tissue types, like epithelial cells, connective tissue, and endothelial cells. If this sequence is impaired or blocked due to genetic or environmental factors, it can lead to clefting. A cleft is an abnormal space or gap in the upper lip, alveolus, or palate that occurs due to failure of completion of fusion processes in the midline during facial development. Previous studies have identified various genetic factors (genes) that can lead to clefting. The most promising candidates amongst them are *FGF/FGFR* (fibroblast growth factor/FGF receptor) signaling genes and *FOX* (forkhead box protein) genes. We investigated the expression of these genes in tissue material obtained from cleft-affected patients. Our results indicate that these genes profoundly affect the pathogenesis and manifestation of clefts, especially by enhancing local site inflammation and fibrosis. Further, they play a vital role in angiogenesis, apoptosis, and cell proliferation.

**Abstract:**

Orofacial clefts affect hundreds of thousands of children worldwide annually and are usually corrected by a series of surgeries extending to childhood. The underlying mechanisms that lead to clefts are still unknown, mainly because of the multifactorial etiology and the myriad of interactions between genes and environmental factors. In the present study, we investigated the role and expression of candidate genes belonging to the *FGF/FGFR* signaling pathway and *FOX* family in tissue material obtained from 12 pediatric patients undergoing cleft correction surgery. The expression was investigated using immunohistochemistry (IHC) and chromogenic in-situ hybridization (CISH) in three cell/tissue types—epithelial cells, connective tissue, and endothelial cells. We found elevated expression of *FGFR1* in epithelial cells while no expression was observed in endothelial cells. Further, our results elucidate the potential pathogenetic role of *FGFR1* in cellular proliferation, local site inflammation, and fibrosis in cleft patients. Along with *bFGF* (also called *FGF2*), *FGFR1* could play a pro-inflammatory role in clefts. Over-amplification of *FGFR2* in some patients, along with *bFGF*, could potentially suggest roles for these genes in angiogenesis. Additionally, increased expression of *FOXE1* (also called *TTF2*) contributes to local site inflammation. Finally, zero to low amplification of *FOXO1* could suggest its potential role in inducing oxidative stress in the endothelium along with reduced epithelial apoptosis.

## 1. Introduction

Congenital cleft lip and palate is one of the most commonly reported birth defects, affecting hundreds of thousands of children worldwide [1]. A cleft is defined as an abnormal space or gap in the upper lip, alveolus, or palate that occurs due to failure of completion of fusion processes in the midline during facial development [2]. Orofacial clefts have been reported to occur as isolated cases or as associated manifestations in over 500 recognized Mendelian syndromes (Online Mendelian Inheritance in Man (OMIM), www.ncbi.nlm.nih.gov/omim/, accessed on 25 March 2021).

Due to its complex etiology, clinical manifestations of clefting are not limited to only dental complications but also encompass speech difficulties, ear infections, feeding problems, as well as behavioral complications [3]. The treatment of such patients, naturally, is also complex, multidisciplinary, long term, exhaustive (both mentally and physically), and generally involves multiple phases of surgical intervention [2,4]. To achieve the optimum results, both in terms of aesthetic restoration and functional normality, it is extremely important that the surgical care is provided at the right age and right time of development [5].

The process of palatogenesis (embryonic development of lip and palate) is a multistep process that begins at the fourth gestational week [6]. It is a very tightly regulated sequalae of events that involves the development, maturation, and fusion of facial processes in the midline [6]. For this fusion to occur, the bilateral palatal shelves must grow beside the tongue and then elevate towards the midline to form the medial edge epithelium (MEE) [7,8]. This is followed by degeneration of the MEE and disappearance of the midline epithelial seam [9]. This programmed cell death of MEE is mediated by the mesenchymal cells (embryonic connective tissue), which ensure that a continuity can be established across the horizontal plate [7]. The mesenchyme in the palate traces its origin to the cranial neural crest (CNC) cells, a subset of neural crest cells (NCCs). During craniofacial development, CNC cells migrate from the lateral ridges of the neural plate to branchial arches [8]. Similarly, part of the epithelium in the palate traces its origin to pharyngeal ectoderm-derived epithelial cells [8,10].

The crosstalk (mediated by cytokines) between the epithelium and the mesenchyme is crucial for normal palatogenesis (especially for degeneration of the MEE) and any disruption in the mesenchyme–epithelial crosstalk eventually leads to clefts of varying severity [6,7,8,9,10]. The crosstalk can be disrupted by various genetic and environmental factors, like maternal smoking, nutrition, alcohol consumption, and so on. Further, defects, either in facial mesenchyme patterning, growth or in epithelium fusion, also result in cleft palate [8]. Occasionally, a fibrous band, known as Simonart’s band, is observed attached to the cleft, suggesting that occlusive epithelial adhesions can also result in clefting [11].

Therefore, it is imperative to study the genes/pathways that regulate or control this crosstalk during palatogenesis in order to completely understand the underlying pathogenesis. Many candidate genes have been suggested to play a role in crosstalk (directly or indirectly) and carry mutations in patients with non-syndromic clefts. While the roles of genes like *IRF6* (interferon regulatory factor 6) [12,13] and *VAX1* (ventral anterior homeobox 1) [14,15] have been confirmed after extensive research, the roles of other genes are yet to be fully substantiated and confirmed. Other promising candidates include the *FGF* (fibroblast growth factor) signaling family genes [16,17,18], *FOX* (forkhead box protein) family genes [19,20], *MSX1* (msh homeobox 1) [21,22], and *BMP4* (bone morphogenetic protein 4) [23].

The *FGF* family includes 18 members that mediate their actions via four distinct receptors (FGFRs). These receptors show differential binding properties and, upon activation, regulate various cellular processes such as cell proliferation, differentiation, apoptosis, and mobility [24,25]. The *FGF/FGFR* family plays a vital role in maintenance of bone homeostasis and mutations in these genes are associated with various congenital bone diseases, including chondrodysplasia syndromes, craniosynostosis syndromes, and syndromes with dysregulated phosphate metabolism [25,26]. *FOX* family genes, on the other hand, consist of transcriptional regulators, divided into 19 classes from *FOXA* to *FOXS* [11], which are involved in the development of various organs, regulation of senescence or proliferation, and metabolic homeostasis [27].

Due to their crucial roles in mesenchymal–epithelial crosstalk and in overall craniofacial development, in the present study we investigated the expression and role of *FGF/FGFR* signaling pathway genes and the *FOX* family genes in orofacial clefts. Further, we investigated the expression of these genes in different tissues/cells—epithelial cells, connective tissue, and endothelial cells. Finally, we investigated their possible roles in subsequent local inflammation, along with possible gene–gene interactions and the role of environmental factors, to allow for better understanding, prediction, and diagnosis of clefts, as well as better treatment modalities.

## 2. Materials and Methods

### 2.1. Profile of Study Participants

In the present study, tissue samples were obtained from 12 pediatric patients (10 male children and 2 female children) who presented for consultation and treatment at the Department of Oral and Maxillofacial Surgery, Institute of Stomatology, Riga Stradiņš University (RSU), Latvia. The tissue for all patients was collected from the site of clefting by the same surgeon. The study was approved by the Research Ethics Committee (REC) of RSU with the approvals dated 22 May 2003, 17 January 2013, and 28 June 2018, in accordance with the 1975 Helsinki Declaration (as revised in 2008). Written informed consent was obtained from all patients (given by the parents) for participation in the study and publication of the study data.

Children in the study were aged between 3 and 18 months at the time of tissue collection and were scheduled for plastic surgery of either bilateral or unilateral clefts. None of the children were previously diagnosed with coexisting genetic syndromes, chromosomal abnormalities, or immune deficiencies. Briefly, mothers of two infants were reported to have threats of miscarriage in pregnancy; two other infants were reported to have parents with histories of smoking; three infants had mothers who used paracetamol in pregnancy and two infants presented with family histories of genetic disorders (however, the children were not affected). Table 1 summarizes the clinical information of the patients.

### 2.2. Data and Sample Collection

Tissue samples were collected immediately after the plastic surgery and fixed for a day in a mixture of 2% formaldehyde and 0.2% picric acid in 0.1 M phosphate buffer (pH 7.2). Next, the samples were rinsed in Tyrode buffer (content: NaCl, KCl, CaCl2·2H2O, MgCl2·6H2O, NaHCO3, NaH2PO4·H2O, glucose) containing 10% saccharose for 12 h followed by paraffin embedment. Samples were registered and assigned randomized sequence tags. Patient identity was not disclosed at any time to the researchers and/or lab assistants, in accordance with protocol. Only patient history (as shown in Table 1) was kept with the sequence tags.

### 2.3. Routine Histological Investigation

In accordance with standard laboratory procedures, 3–4 µm of serial tissue sections were prepared from the solidified paraffin block for histological staining and immunohistochemistry (IHC). Tissue sections placed on the slides were kept at 56 °C for 20–60 min in a thermostat. De-paraffinization of the sections was undertaken using xylene solution and 96% ethanol alcohol. Hematoxylin and eosin staining (Mayer’s; Bio Optica Milano, Milan, Italy) was undertaken using standard procedure.

Slides were dehydrated with ethanol and clarified with carboxylol and xylene. Finally, a drop of histological Pertex glue (Histolab Products AB, Askim, Sweden) was applied and slides were covered with a cover glass. Slides were visualized using brightfield light microscopy with a Leica DC 300F camera microscope (Leica DM500RB; Leica Biosystems Richmond, Richmond, IL, USA).

### 2.4. Immunohistochemistry (IHC)

De-paraffinized, washed, and cleared tissue sections were rinsed with TRIS buffer (Diapath, Martinengo, Italy) for 10 min followed by boiling in EDTA buffer in a microwave for 10 min. The tissue samples were cooled to 65 °C and then placed again in TRIS wash buffer. Endogenous peroxidase was blocked with 3% peroxidase (Dako, Naestved, Denmark). *FGF* basic (ab16828, working dilution 1:200, rabbit, Abcam, Cambridge, UK), *FGFR1* (orb38277, working dilution 1:50, rabbit, Biorbyt Limited, Cambridge, UK), and *FOXE1* (ab5080, working dilution 1:500, goat, Abcam, Cambridge, UK) antibodies were used for biotin–streptavidin immunohistochemistry.

All antibodies were diluted with antibody diluent (Cell Marque^TM^, Rocklin, CA, USA). Incubation with primary antibody was performed for 2 h followed by washing in TRIS wash buffer. The HiDef DetectionTM HRP polymer system (Cell Marque^TM^, Rocklin, CA, USA) was used for rabbit antibodies as per the manufacturer’s guidelines. The ImmunoCruz^TM^ ABC staining system was used for goat antibody (Santa Cruz Biotechnology, Dallas, TX, USA) as per the manufacturer’s guidelines. Tissue sections were incubated with biotin-containing secondary antibody for 30 min and rinsed again for 10 min in TRIS wash buffer, followed by another round of incubation and washing with biotin-containing tertiary antibody and TRIS wash buffer.

The tissue sections were then coated with the DAB+ chromogenic liquid using a DAB Substrate Kit (Cell Marque^TM^, Rocklin, CA, USA) and incubated at room temperature for up to 10 min to obtain brown staining of immunoreactive structures. The sections were then washed in distilled water and contrast-stained with hematoxylin for 2 min. The antibody-treated tissue material was dehydrated with ethanol solutions and clarified with carboxylol and xylene. The slides were prepared and viewed under a light microscope (as described previously). Negative and positive IHC controls were prepared for each sample in the study.

### 2.5. Chromogenic In-Situ Hybridization (CISH)

CISH is a relatively new technique that utilizes a chromogen-labeled DNA probe which is often visualized using peroxidase reaction. The technique is based on the principle of subtractive hybridization. The technique allows for simultaneous assessment of tissue morphology and CISH signals and is standardized with complete kits, thereby eliminating the need to perform the more expensive fluorescent microscopy [28,29]. Due to easy interpretation of results and the technique’s superiority when compared with IHC, we decided to detect the signals of the candidate genes using CISH.

CISH was performed using ZytoDot 2C CISH Implementation Kit (ZytoVision GmbH, Bremerhaven, Germany). Probes of *FGFR1, FGFR2*, and *FOXO1* were used in this study. Pretreatment was performed using standard laboratory methods. Denaturation and hybridization were undertaken using 10 μL of each probe placed on each pretreated specimen with a pipette. Slides were covered with an 18 mm × 18 mm coverslip and placed on a hot plate for 5 min at 79 °C, then transferred to a humidity chamber and hybridized overnight at 37 °C.

To proceed with the detection process, the coverslips were removed by submerging the slides in SSC wash buffer followed by TBS wash buffer. Then, the slides underwent the next steps in the CISH procedure as per the manufacturer’s guidelines. Slides were transferred into a staining jar and washed for 2 min under cold running tap water. Dehydration was undertaken with 100% ethanol and the slides were then incubated in xylene. The coverslips were re-attached while avoiding air bubbles and the slides visualized under a light microscope.

Under the microscope, red-colored dots indicated control, whereas green-colored dots indicated target. After counterstaining the nucleus with a nuclear dye, hybridized probe fragments were visualized. Two signals per probe were expected to appear in the cell nuclei of normal cells in interphase or metaphase or in the nuclei of cells without aberrations in the examined chromosomes.

### 2.6. Visualization and Statistical Analysis

The images obtained using microscopy were analyzed with Image Pro Plus 6.0 (Media Cybernetics, Rockville, MD, USA). Cells with nucleus/cytoplasm marked brown in the IHC reaction were considered as immunoreactive, i.e., as showing immunopositivity. For CISH, the signals from the green probes in the cells/nuclei were evaluated. Epithelium, Connective Tissue, and wall of mucosal microcirculation blood vessels (i.e., Endothelium) were assessed. Semi-quantitative counting was undertaken by two independent morphologists in at least five randomly selected vision fields, each at 400× magnification, for each tissue section in order to quantify the immunoreactive and probe-containing cells (Table 2) [30,31].

Statistical analysis was performed using non-parametric Kruskal–Wallis ANOVA with appropriate post hoc tests and Bonferroni correction for inter-group comparison. Spearman’s Rho was used for correlation analysis. The data were stored and analyzed using MS Excel (MS Office 365) and SPSS v26.0 (IBM Corp., Armonk, NY, USA). For statistical analysis, the numbers of “+” values were considered as equivalent to absolute whole numbers (e.g., “+” corresponded to 1; “++” corresponded to 2, and so on). Statistical significance was set at *p* < 0.05.

## 3. Results

### 3.1. Immunohistochemistry Analysis

**Figure 1:**
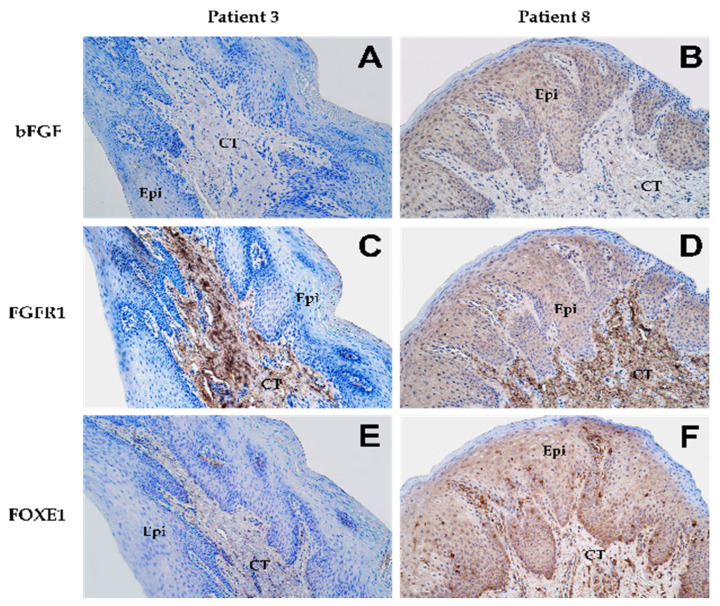
Expression patterns of various proteins as shown by immunohistochemical staining in lip mucosal tissue obtained from patient 3 (**A**,**C**,**E**) and patient 8 (**B**,**D**,**F**) at 200× magnification. “Epi” denotes epithelium while “CT” denotes connective tissue. Expression of *bFGF* in the epithelium shown by (**A**) none of the cells (0) and (**B**) a moderate number of cells (++). Connective tissue in both patients (**A**,**B**) showed no expression (0) of *bFGF*. Expression of *FGFR1* in the epithelium shown by (**C**) none of the cells (0) and (**D**) a moderate number of cells (++). In connective tissue, both patients (**C**,**D**) presented numerous cells (+++) showing expression of *FGFR1*. Expression of *FOXE1* in the epithelium shown by (**E**) none of the cells (0) and (**F**) numerous cells (+++). However, in connective tissue, patient 3 (**E**) showed few positive cells (+) while patient 8 (**F**) showed numerous positive cells (+++) for *FOXE1*.

As shown in Figure 1 and Table 3, low to moderate numbers of epithelial cells in the tissue demonstrated *bFGF*, *FGFR1,* and *FOXE1* protein expression (mean IHC semi-quantitative grade: 1.2+, 1.4+, and 1.5+, respectively). All three proteins, however, showed high variation in expression amongst the patients, as evidenced by the high coefficient of variation (CV%). In the connective tissue, expression of *bFGF* and *FGFR1* was undetectable, with only a few cells showing a positive reaction (except in patients 3 and 8). In contrast, *FOXE1* expression was seen in numerous cells, with small variation amongst the patients (CV% = 29%). 

None of the endothelial cells showed a positive reaction for *FGFR1* protein in any of the patients while *bFGF* protein was evident only in a few cells. A high abundance of cells showed positive reaction for *FOXE1* in the endothelium (mean IHC semi-quantitative grade: 3.75+). Further, expression of *FOXE1* protein was also found to be the least variable (low CV%) amongst patients, indicating its near-universally high positive reaction in endothelial cells.

**Figure 2:**
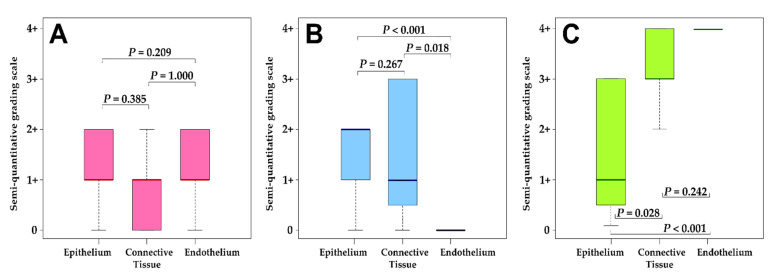
Distribution of semi-quantitative grading for IHC staining for (**A**) *bFGF*, (**B**) *FGFR1*, and (**C**) *FOXE1*. The adjusted *P* values indicated were obtained using the post hoc tests from the Kruskal–Wallis ANOVA test. An interpretation of the grading scale is shown in Table 2.

Kruskal–Wallis ANOVA post hoc analysis revealed that there were significant differences in the number of cells expressing *FGFR1* and *FOXE1* proteins amongst the three cell types (Figure 2B,C). A significantly greater number of both connective tissue and epithelial cells showed positive reactions for *FGFR1* compared to the endothelial cells (*p* = 0.018 and < 0.001, respectively). For *FOXE1*, a significantly higher number of epithelial cells showed positive reactions compared to both connective tissue and endothelial cells (*p* = 0.028 and < 0.001, respectively). No significant differences were obtained for the number of cells positive for *bFGF* protein expression amongst the tissue types (Figure 2A).

### 3.2. Chromogenic In-situ Hybridization Analysis

**Figure 3:**
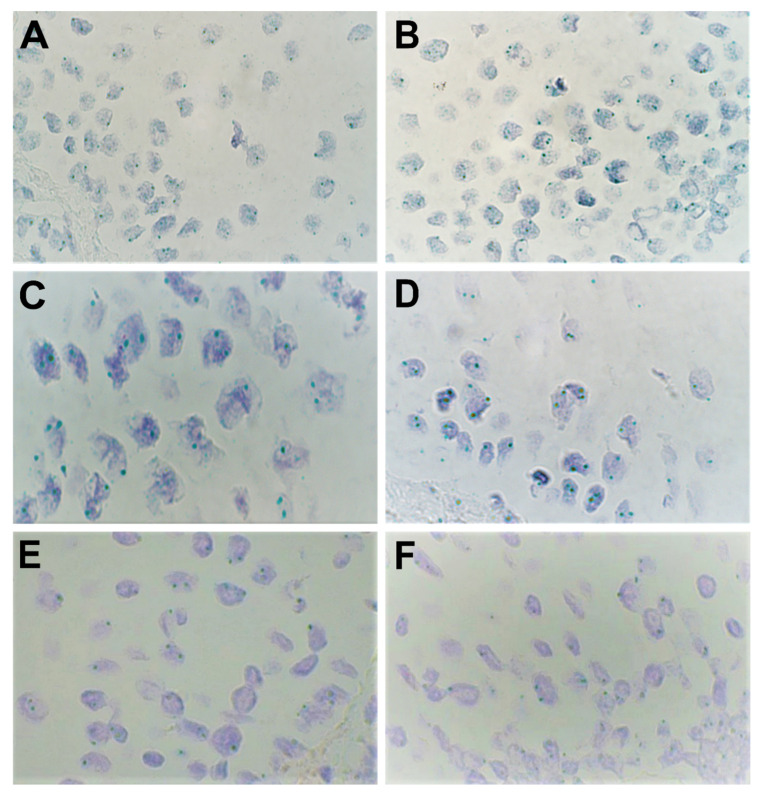
Expression of various genes as shown by chromogenic in-situ hybridization (CISH) in lip mucosal tissue at 1000× magnification (visualized using immersion oil). Amplification was visualized through the number of green signals (dots) per nucleus per cell. (**A**,**B**) Moderate amplification (++) was found for levels of *FGFR1* in the epithelium in patients 2 and 5, respectively. (**C**) Weak amplification (+) and (**D**) no amplification (0) were found for levels of *FGFR2* in the epithelium in patients 9 and 1, respectively. (**E**,**F**) No amplification (0) was found for levels of *FOXO1* in the epithelium in patients 8 and 12, respectively.

As shown in Figure 3 and Table 4, zero to low levels of amplification were observed for *FGFR1* and *FGFR2* in the epithelial tissue (mean CISH semi-quantitative grade: 0.67+ and 0.75+, respectively). However, the level of amplification was not uniform across the patients and varied greatly, indicating the role of other potential influencing factors (CV% > 100%). In the connective tissue, *FGFR1* demonstrated a low level of amplification while *FGFR2* demonstrated no amplification.

In the endothelium, almost no amplification was noted for both *FGFR1* and *FGFR2*. No amplification was detected for *FOXO1* in any of the three tissues. Further, the Kruskal–Wallis test showed no significant difference in amplification levels of *FGFR1* and *FGFR2* between the three cell types (*p* = 0.0583 and 0.0581, respectively). Significant differences in amplification levels amongst the cells were noted for *FOXO1* (*p* = 0.0415).

### 3.3. Correlation Analysis.

**Figure 4:**
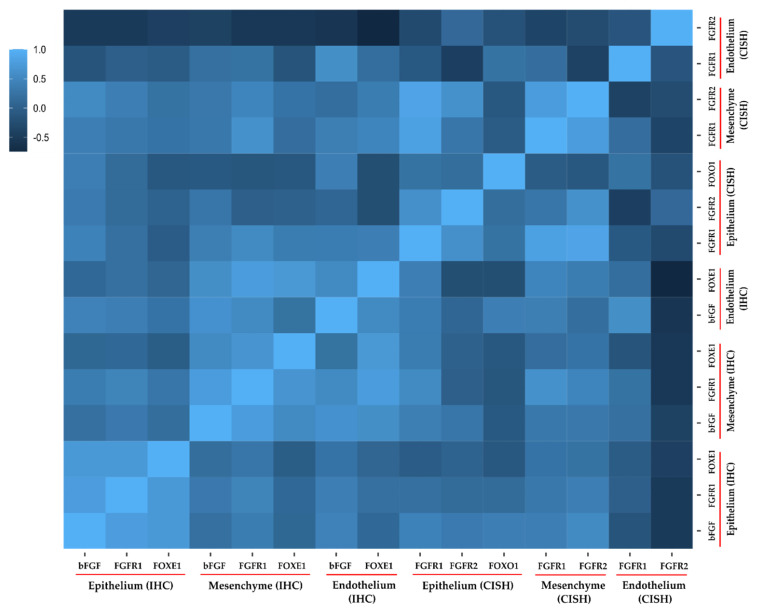
Correlation heatmap for different genes/proteins studied in the present study. The legend shows the correlation strength as determined using Spearman’s rho (ρ). Mesenchyme refers to connective tissue. A standard interpretation scale was used, i.e., 0.01 to 0.19 indicated no correlation; 0.20 to 0.29 indicated a weak correlation; 0.30 to 0.39 indicated a moderate correlation; 0.40 to 0.69 indicated a strong correlation; and ≥0.70 indicated a very strong correlation. IHC denotes immunohistochemistry; CISH denotes chromogenic in-situ hybridization.

With regard to the immunohistochemistry results, *FGFR1* in connective tissue showed a significant (*p* < 0.05) and very strong positive correlation with *bFGF* in connective tissue (ρ = 0.762) and *FOXE1* in endothelial cells (ρ = 0.771), while a strong positive relationship was noted with *FOXE1* in connective tissue (ρ = 0.668). As shown in Figure 4, in the epithelium all three proteins showed very strong significant positive correlations with each other (*bFGF–FGFR1* with ρ = 0.775; *bFGF–FOXE1* with ρ = 0.725; and *FOXE1–FGFR1* with ρ = 0.720). A strong positive correlation was observed for *bFGF* in connective tissue and the endothelium (ρ = 0.645). A very strongly association was found for *FOXE1* in connective tissue and the endothelium (ρ = 0.708).

In regard to the chromogenic in-situ hybridization results, a strong to very strong positive correlation was observed between *FGFR1* in epithelial cells and *FGFR2* in both epithelium and connective tissue, respectively (ρ = 0.596 and 0.855, respectively). Further, *FGFR1* in the epithelium was also very strongly correlated with *FGFR1* in connective tissue (ρ = 0.821). A similar observation was noted for *FGFR2* in the epithelium and connective tissue (ρ = 0.637). In connective tissue, both *FGFR1* and *FGFR2* were also very strongly associated (ρ = 0.767). Utilizing both the CISH and IHC results, a very strong and significant negative correlation was observed between *FOXE1* and *FGFR2* in endothelial cells (ρ = −0.739). Similarly, a strong positive association was noted for *bFGF* and *FGFR1* (CISH) in endothelial cells (ρ = 0.590).

## 4. Discussion

A multifactorial pathoetiology combined with high incidence and translated downstream high socio-economic burden makes orofacial clefts of particular interest to various research groups working to elucidate the factors/cellular pathways that play a role in pathogenesis of clefts. Understanding these interactions, which can range from gene–gene (GxG) to gene–environment (GxE), is crucial for creating models and systems that can aid in predication, diagnosis, prognosis, and treatment of cleft pathology. Over 70% of cases of cleft lip and palate are non-syndromic while the remainder comprise of syndromic cases and of those syndromes that usually arise secondary to chromosomal or teratogenic effects [11]. The etiology of syndromic forms is traceable by genetic analysis, which reveals the underlying genetic mutation responsible. However, in cases of isolated, non-syndromic clefts, the gene–environment interactions (GxE), which are thought to be the main reasons for clefting, are difficult to confirm. This warrants a search for possible gene–gene interactions (GxG) and their interactions with environmental teratogens in order to isolate the underlying cause of clefting [32].

### 4.1. Fibroblast Growth Factor Receptor 1 (FGFR1)

The *FGF/FGFR* signaling genes are expressed in a spatiotemporally specific manner in the palatal tissue and constitute a directional regulatory axis between the stromal and epithelial compartments [8,17]. *FGFR1*, which is highly expressed in the CNC-derived connective tissue (mesenchyme) in the palatal shelf, is usually responsible for mediating epithelium-to-mesenchyme signaling while *FGFR2* mediates the reciprocal communication (hence *FGFR2* is more abundant in the epithelium). In a study on mice conducted by Wang et al. [8], the authors demonstrated that ablation of *FGFR1* in NCCs delayed (not inhibited) cellular proliferation of both the mesenchyme and epithelium and impeded development of medial nasal processes. Further, they reported impeded elevation of palatal shelves prior to midline fusion (a key event in normal palatogenesis). Additionally, *FGFR1* was also shown to play an important role in controlling MEE degeneration during palate fusion [8]. In contrast, it has also been shown that increased expression of *FGFR1* leads to increased cellular proliferation in the palate shelves and cleft palate [33].

Therefore, both excess and deficiency of *FGFR1* signaling leads to clefting and a precise balance in protein and gene expression are needed for normal palatogenesis. In the endothelium, *FGFR1* plays a key role in inhibiting the endothelium–mesenchyme transition (EndMT) via the inhibition of TGFβ/Smad signaling pathway, thereby preventing tissue fibrosis and maintaining a normal state of vascularity [34]. Further, loss of endothelial *FGFR1* and *FGFR2* has been shown to result in impaired neovascularization after injury in adult mice [35]. *FGFR1* has also been shown to be widely expressed in the myofibroblasts of injured palates, suggesting that *FGFR1* signaling is also important for palate repair during injury [8,36]. Finally, it has been demonstrated that disruption of *FGFR1* signaling reduces local inflammation by restraining activation of the NF-κB signaling cascades [37,38]. Clearly, *FGFR1* plays a key role in maintaining normal development processes and in preventing fibrosis and inflammation.

Overall, our IHC and CISH results indicate that more *FGFR1* receptors are present in epithelial cells, followed by connective tissue cells, and none in the endothelial cells. The presence of elevated expression of *FGFR1* in cleft epithelium thus, leads to increased cellular proliferation that may lead to clefting and local site inflammation. This is in line with our previous studies in which we demonstrated moderate expression of proliferation marker Ki67 in epithelial cells from cleft tissue (compared with no Ki67 expression in control samples) [39].

Further, the fact that *FGFR1* was not detected in the endothelium indicates its potential role in promoting fibrosis [34]. This finding potentially also correlates clinically with the post-operative complications, like slow healing and hypertrophic scars, that are frequently reported in cleft patients [40]. Further, elevated expression of *FGFR1* leads to a significant decrease in TGFβ1 expression, a potent anti-inflammatory and immunosuppressive factor, which brings our findings in line with those of our previous study [41].

### 4.2. Fibroblast Growth Factor Receptor 2 (FGFR2)

*FGFR2*, another associated FGF receptor, is primarily expressed in the developing palatal epithelium that binds *FGF10*, a factor known to be localized in the adjacent underlying mesenchyme [42]. Deficiency of either *FGFR2* or *FGF10*, or both, leads to clefting and a thin epithelium due to the severe reduction in cell proliferation [42]. Since *FGF10/FGFR2* signaling affects the epithelium, there is a lack of *FGFR1*-mediated reciprocal signaling from the epithelium, which consequently leads to proliferation defects in the mesenchyme [42]. Further, loss of *FGFR2* has been shown to compromise the organization of the rugae (the thickened lines on the secondary palate) [43]. *FGFR2* has two main epithelial isoforms, namely *FGFR2b* and *FGFR2c*. While *FGFR2b* has been associated with tumor suppression, amplification of *FGFR2c* has been linked with various epithelial tumors [44,45]. Further, it has been shown that an abnormal switch from *FGFR2b* to the *FGFR2c* isoform could be the main triggering event leading to epithelium-to-mesenchyme transition (EMT) in normal human keratinocytes [46].

In a study on human keratinocyte cell line HaCaT, Ranieri et al. concluded that increased expression of *FGFR2c* leads to morphological and cytoskeletal changes, gene reprogramming, and invasive behavior, reminiscent of type III EMT (seen in carcinogenesis in which epithelial cells may completely lose their vestiges and become fully mesenchymal) [45]. Further, higher expression of *FGFR2b* has been associated with physiological type II EMT (seen in adult tissue regeneration). Since our *FGFR2* CISH probe could not distinguish between the two isoforms, it was difficult to conclude, for patients 2, 5, and 11, who showed moderate amplification, and patients 7 and 3, who showed low amplification in the epithelium, which splicing isoform was more abundant and, hence, the type of EMT. Therefore, we can conclude that *FGFR2* in general demonstrates a specific expression pattern and that more intensive studies investigating the role of *FGFR2* isoforms are needed to further our understanding in predicting the clinical course of clefts.

### 4.3. Basic Fibroblast Growth Factor/Fibroblast Growth Factor 2 (bFGF/FGF2)

*bFGF* has been described as a biological mediator that regulates connective tissue cell migration, proliferation, and synthesis of intracellular proteins and extracellular matrix [47,48]. It has been shown to induce angiogenesis via VEGF (vascular endothelial growth factor) in vitro [49], while in vivo it has been demonstrated to accelerate the healing process [50]. Additionally, Choi et al. found that *bFGF* suppressed collagen type I generation and subsequent scar tissue formation [48]. Like *FGFR2b*, *bFGF* has also been associated with the potential to accelerate type II EMT, thereby promoting wound closure [51]. Elevation of *bFGF* levels in our patients was in line with previous findings in which *bFGF* was reported to be increased in concentration in the serum and affected tissue of patients with chronic inflammation and rheumatoid arthritis [52,53]. *bFGF* has been shown in vivo to enhance the recruitment of monocytes, T cells (due to elevated IFN-γ (interferon gamma)), and PMN (polymorphonuclear) cells in response to elevated TNF-α (tumor necrosis factor alpha) and IFN-γ [52]. These findings are in line with our previous study in which we reported elevated TNF-α and IFN-γ levels [41], indicating that pro-inflammatory cytokines mediate their inflammatory effects via elevation of *bFGF* in cleft tissue. Thus, our findings also indicate that *bFGF*, along with other factors, induces inflammatory and immune cell recruitment, which chronically leads to an angiogenic environment (supported by results showing elevated *bFGF* protein expression in endothelium from IHC) [41,52].

### 4.4. Potential Roles Mediated By FGF/FGFR Family Genes in Cleft Lip/Palate Pathogenesis

It has been reported that the *bFGF/FGFR1* axis can exert an anti-inflammatory response in astrocyte-mediated neuroinflammation, especially after infrasound exposure [54]. However, other authors have reported that the axis exerts a pro-inflammatory effect [38,40,55]. Hence, in the case of orofacial clefts, we postulate that this axis indeed exerts a pro-inflammatory effect. The elevated expression of *FGFR1* and *bFGF* and the high correlation between them in the epithelium, connective tissue, and endothelium cells supports our hypothesis. The correlation indicates a positive feedback loop between them, as previously it has been demonstrated that *bFGF* can upregulate the expression of *FGFR1* on astrocytes [54]. It has also been shown that endothelial cells can be stimulated to produce and release *bFGF* by pro-inflammatory mediators like IFN-γ in combination with IL-2, IL-1β, and nitric oxide (NO) [56,57]. This creates a vicious cycle whereby damaged mucosal tissue (due to improper palatal closure, shear stress, or shock) secretes IFN-γ, TNF-α, IL-2, and so on, which upregulate *bFGF*, which then upregulates *FGFR1* protein expression that then recruits PMNs, T cells, and so on. These cells, in turn, secrete more *bFGF* [58,59] which sustains inflammation (via *FGFR1*) and angiogenesis (via *FGFR2*) at the cleft site, leading to chronic inflammation and fibrotic changes and tissue scarring.

We expected that in almost half of our patients—those with no detectable *FGFR2* amplification (patients 1, 4, 6, 8, 9, 10, and 12)—there would be low or zero amplification/protein levels for *FGFR1* since the crosstalk was impaired. However, our findings regarding *FGFR1* amplification and the moderate number of cells expressing *FGFR1* protein confirm the possible amplifying role of *bFGF* in *FGFR1* expression and the importance of these receptors in the pathogenesis of clefts. Additionally, hypoxia has been associated with the pathogenesis of cleft lip and palate; however, the exact mechanism is not yet known. Recently, polymorphisms of the *HIF-1A* (hypoxia inducible factor 1A) gene were ruled out as a cause of clefting [60]. We propose that hypoxia-induced clefting is probably rather related more directly with the *FGF/FGFR* signaling pathway (this needs further validation). Conte et al. found that, in the early hypoxia phase, *bFGF* upregulation does not occur at the mRNA level but at the protein level. This upregulation (in vascular smooth muscle cells) is due to increased ribosomal translational activity via IRES (internal ribosome entry site, a transcriptional regulator) rather than HREs (hypoxia response elements), which are used by *HIF-1A* to regulate *bFGF* expression [61]. However, in later stages of hypoxia, *bFGF* induces *HIF-1A* protein expression which then regulates *bFGF* expression (via HREs), thereby creating an autocrine amplification loop [62].

Interestingly, hypoxia along with cell damage and fluid/plasma protein exudation can also manifest as an effect of clefting in tissue due to chronic inflammation. Hypoxia can increase the sensitivity of endothelial cells via increased HSPG (heparan sulfate proteoglycan) synthesis in endothelial cells [63] along with increased *bFGF* production by vascular pericytes [64]. Finally, regarding angiogenesis induced by the *bFGF/FGFR2* pathway, although autocrine amplification has been reported [65], its implications and effects are relatively unknown. Additionally, negative regulators that bind excessive *bFGF* have also been reported. *PF4* (platelet factor 4) and *PTX3* (pattern recognition receptor pentraxin 3, synthesized in response to TNF-α and IL-1β) both bind *bFGF*, leading to its decreased interaction with *FGFR1/2* [66,67]. In fact, the roles of *PTX3* and *PF4* in cleft lip and palate have never been reported before and we suggest this as a potential new avenue for investigation since they modulate the binding of *bFGF* with its receptors.

### 4.5. Forkhead Box Protein E1/Thyroid Transcription Factor 2 (FOXE1/TTF2)

Also known as thyroid transcription factor 2 (*TTF2*), *FOXE1* is usually expressed in later stages of development in tissues derived from pharyngeal arches and the pharyngeal wall, including the thyroid, tongue, epiglottis, palate, and esophagus [68]. Further, it has been shown to be co-expressed with *Shh* (sonic hedgehog) in these tissues, usually around the same time, and to mediate the epithelial–mesenchymal crosstalk [68]. Recently, analysis revealed that amplification of *FOXE1* affects *MSX1* and *TGF-β3* downstream in the exertion of its action in the cleft tissue [69]. Interestingly, both these targets are found to be abundant at the sites where epithelial–mesenchymal interactions take place, including the cellular primordia involved in craniofacial morphogenesis [69,70]. To add to this, *MSX1* is another commonly associated gene with isolated non-syndromic clefts [21,22]. *TGF-β3*, on the other hand, is expressed in the MEE of pre-fusion shelves, with its expression decreasing shortly after the midline epithelial seam is formed [69,71].

It has been shown in thyroid tissue that *FOXE1* regulates the activity of *NR4A2* (nuclear receptor subfamily 4 group A member 2). The authors showed that there is an indirect regulation between *FOXE1* and *NR4A2* whereby silencing of *FOXE1* leads to downregulation of *NR4A2* [72]. The gene *NR4A2* belongs to the family of ligand-independent early response genes, which are involved in proliferation, apoptosis, and inflammation and have been demonstrated to enhance migration of mesenchymal stromal cells [73]. Additionally, it has been shown that TGF-β1/2 significantly downregulates the expression of *NR4A2* in palatal mesenchymal cells [74]. *NR4A2* has also been shown to be induced by TNF-α, IL-1β, and VEGF in synoviocytes, chondrocytes, endothelial cells, and immune cells [75,76]. Elevated expression of *FOXE1* in our patients and the fact that VEGF can be induced by elevated *bFGF* (as seen in our patients), coupled with elevated TNF-α and downregulated TGF-β1 in cleft patients [41], suggests that local site inflammation is alternatively maintained in part by upregulation of the *FOXE1*/*NR4A2* pathway in cleft patients.

### 4.6. Forkhead Box Protein O1 (FOXO1)

*FOXO1*, another transcriptional regulator, has been shown to play a crucial role in palatogenesis. It is needed for transcriptional promotion of the pro-apoptotic Fas ligand (FasL)/caspase-3 pathway in MEE cells, which paves the way for correct palate fusion [77]. However, for *FOXO1* to function it needs to undergo acetylation by p300 in the presence of *BAG6* (Bcl-2-associated anthanogene 6) [78]. *BAG6* has also been recently described as another candidate gene for non-syndromic clefts. Non-detection of *FOXO1* amplification in our patients indicates upstream signaling impairment which leads to clefting. *BAG6* has also been shown to induce TGF-β3 transcriptional target expression [79], which shows the complex web of interactions taking place in cleft tissue. *FOXO1* also plays a crucial role as a gatekeeper in endothelial cells. It restricts endothelial growth, thereby lowering endothelial metabolism and supporting the function of endothelial cells [80]. This helps the cells to consume less energy, nutrients, and O2, thereby reducing oxidative stress in endothelial cells. Deletion of *FOXO1* has been shown to cause a profound increase in endothelial proliferation that interferes with coordinated sprouting, thereby causing hyperplasia and vessel enlargement [80]. Hence, no to low amplification of *FOXO1* (as seen in our patients) leads to increased oxidative stress and damage in the cells, leading to promotion of inflammatory changes in the tissue.

### 4.7. Clinical Diagnostic Techniques and Advances for Cleft-Affected Patients

Cleft lip and/or palate have been reported to be associated with aplasia of the salivary glands, which leads to xerostomia, multiple dental caries, and early tooth loss [81,82]. The characteristics of the tissue replacing the defective sites can also be evaluated without surgical intervention using ultrasound, CT, MRI, or FNA (fine needle aspiration) [83]. Since salivary glands and the oral epithelium share the same ectodermal origin, it has been demonstrated that genes involved in clefting are also expressed in ductal epithelium during embryogenesis [84,85,86]. Further, it has been reported that the absence of *FGFR2b* is associated with an absence of salivary glands in mouse models [87]. Since more than half of our patients did not show detectable amplification of *FGFR2b*, it would be interesting to further study and investigate the salivary gland tissue in cleft-affected patients.

Further, with the advent of new modern technologies, like microtomography and peripheral quantitative computed tomography (pQCT), it is easier than ever to precisely map and reconstruct in 3D the morphology of the oral structures, both quantitatively and qualitatively [88]. Such techniques could, in the future, have the potential to aid and predict the outcome and severity of clefting, as well as predict the outcomes of treatments, i.e., post-operative complications like inflammation, scarring, and so on. Other digital techniques, like CAD/CAM technology and intra-oral digital scanners, can aid in the appropriate diagnostics and management of cleft-affected patients [89,90].

These techniques are more efficient compared to conventional impression modeling techniques since they get rid of the need to send impression models physically. A single electronic file can be sent to laboratories for investigation, thereby saving time, resources, and space. They also eliminate distortions as well as volumetric variations related to impression material [91,92].

### 4.8. Surgical Management of Cleft-Affected Patients

The main aim of surgical management is the early reconstruction of normal anatomy to allow for physiologic growth of the midface structures and enable children to develop undisturbed mastication, speech, hearing, and esthetics [93,94]. The generally accepted time for intervention has historically been reported as 10 weeks (2.5 months) after birth, since earlier intervention risks increased chances of post-operative complications during the neonatal period [95]. Advances in neonatal care and technology have made it possible to provide surgical care even at earlier stages (within the first 28 days post-birth) [96]; however, no significant differences have been reported in terms of cosmetic attraction or the success of the surgical outcome, even when the surgery is not done in the neonatal period [97]. For patients with clefting of the hard palate, the optimal time of surgery is within 18 months post-birth, since repair at later stages, i.e., beyond three years of age, has been associated with severely restricted language development and stereotyped cleft speech characteristics [95,98].

Cleft-affected patients usually undergo reconstruction of the alveolar ridge (gingiva-peri-osteoplasty) in combination with closure of the lip. However, despite this, 60–80% of patients require a second bone graft or sinus lift to allow for implant placement [93]. In these patients, apart from plastic surgery, it is important to preserve the bone volume along with augmentation of the vertical aspect of the bone. This could be followed by localized sinus elevation with minimal surgical trauma, thereby increasing the available bone for implant placement [99,100,101]. The crestal bone is displaced toward the sinus floor and the apical portion of the implant is placed in the augmented space [99,100,101]. Additionally, human maxillary sinus membrane tissue represents a potential source of multipotent mesenchymal stem cells that can promote a natural healing process [99,102,103]. Further, since the incidence of missing teeth in cleft patients is close to sixfold higher than in non-cleft patients, insertion of endosseous dental implants to replace missing teeth is greatly preferred [93,104,105,106]. Finally, maintenance of oral hygiene is of high importance in such patients to prevent the inserted implant from failing and to prevent systemic spread of infection from the oral cavity [107,108,109].

### 4.9. Relevance and Limitations of the Present Study

The main aim of the present study was to explore and understand the expression levels of *FGF/FGFR* and *FOX* family proteins in tissue from cleft-affected children. Firstly, the expression analysis of these genes allows an understanding of their role in promoting post-operative complications, like scarring, inflammation, and fibrosis. Secondly, since cleft lip and palate is a hereditary condition, gathering knowledge about the chances of the gene being passed on in a family and the nature and severity of the condition caused by the causative genes is inevitably a genuine concern for affected families. Hence, evaluation of such genes enables us to be better positioned to predict the heritability and the course of the manifestation of the clefting. Thirdly, since humans are diphyodonts (i.e., have two sets of dentitions—primary and permanent), it is of utmost importance to understand the gene expression of these genes in cleft-affected tissues, since 60–80% of cleft patients regularly require more than two rounds of corrective surgery and the incidence of missing teeth in cleft children is nearly six times higher than in non-cleft children [93,104,105,106].

Nonetheless, the present study has its limitations, including the limited number of patients studied and the lack of control samples. However, we must point out that the number of studies on human tissue is limited due to the non-availability of tissue material. This is understandable, especially since tissue material needs to be taken during surgery, which happens at a very tender age and is accompanied by genuine parental concerns. Further, decisions regarding procurement of tissue from patients are made with the best interests of patients in mind (if the obtainment of tissue is possible at all). Finally, we cannot at this stage predict or state whether neonatal gene expression resembles embryonic gene expression, especially at the time of palatogenesis. This is firstly because of technological limitations and secondly because of ethical guidelines prohibiting experimentation on human embryos. However, studies in rat models suggest that, during normal palatogenesis, expression of the studied genes, like *FGFR1* and *FGFR2*, is elevated before fusion of the palate but ceases to exist after palatal plate fusion and is exhibited only by MEE cells [110]. Since it is known that MEE cells eventually degenerate due to epithelial–mesenchymal crosstalk, it can be naturally expected that expression of these genes also ceases to exist in palatal tissue. However, detection of such genes (at higher levels of expression) in palatal tissue in our samples makes us to postulate that the palate failed to fuse in these cases (and hence that the patients had clefting); thus, is the closest representation of gene expression during the events of palatal fusion. We suggest that future studies should investigate phenotypic and molecular data from animal models to further bolster our findings.

## 5. Conclusions

The complex myriad of interactions between various factors and environmental teratogens discussed above needs more investigation and in-depth studies. The most significant findings of the present study can be summarized as follow:

1. Elevated expression of *FGFR1* in cleft epithelium indicates its role in mediating cellular proliferation and local site inflammation. No to low expression in the endothelium indicates its role in fibrosis. Coupled together, this indicates that *FGFR1* expression can help in predicting the sequalae and intensity of post-operative complications like scarring.
2. *bFGF* (or *FGF2*) elevation may induce local site inflammation (via *FGFR1*) which chronically leads to creation and promotion of an environment suitable for angiogenic activity (via *FGFR2*). Additionally, over-amplification of *FGFR2* in some patients points to its possible disordered role in epithelial–mesenchymal transition in cleft patients.
3. High expression of *FOXE1* possibly exerts a pro-inflammatory effect via involvement of the *NR4A2/VEGF* pathway (also induced by *bFGF*), while the lack or low level of amplification of *FOXO1* can lead to retention of the midline epithelium coupled with increased endothelial oxidative stress and tissue inflammation.

## Figures and Tables

**Table 1:** Profile of study participants and clinical diagnosis.

Patient Number	Age (in Months)	Gender	Clinical Diagnosis *	Material Collected	Remarks
1	3.5	M	Cheilognathouranoschisis sinistra	Lip mucosa	Mother reported use of paracetamol during pregnancy; father was smoker and partially alcoholic. Epilepsy in the family tree. Child was born overweight.
2	4	M	Cheilognathouranoschisis sinistra	Lip mucosa	There was a reported threat of miscarriage in the 36th gestational week; history of clefts in the family tree.
3	4	F	Cheilognathouranoschisis dextra	Lip mucosa	-
4	4	F	Cheilognathouranoschisis sinistra	Lip mucosa	Born in the 42nd gestational week; mother reported use of paracetamol during pregnancy.
5	4	M	Cheilognathouranoschisis sinistra	Lip mucosa	Born in the 41st gestational week; mother reported use of paracetamol during pregnancy.
6	4	M	Cheilognathouranoschisis dextra	Lip mucosa	History of arrhythmogenic right ventricular dysplasia (ARVD) during the first trimester; mother reported use of Neuromidin, Ibumetin, and Theraflu.
7	4.5	M	Cheilognathouranoschisis sinistra	Lip mucosa	History of Down syndrome in the family tree.
8	5	M	Cheilognathouranoschisis sinistra	Lip mucosa	History of clefts in the family tree; mother reported use of Amoxiclav during pregnancy.
9	8	M	Cheilognathouranoschisis sinistra	Lip mucosa	Both parents were regular smokers.
10	13	M	Cheilognathouranoschisis bilateralis	Lip mucosa	The child had multiple anomalies, including heart failure.
11	4	M	Cheilognathouranoschisis sinistra	Vomer mucosa	History of heavy toxicosis during the pregnancy; there was a threat of miscarriage in the 36th gestational week.
12	18	M	Cheilognathouranoschisis sinistra	Vomer mucosa	Mother was reported to suffer from high emotional stress.

* Clinical diagnosis is provided in Latin; cheilognathouranoschisis—cleft lip, alveolar ridge, and palate; sinistra—left; dextra—right; bilateralis—bilateral.

**Table 2:** Semi-quantitative grading scale used in the present study.

Assigned Value	In-Lab Criteria Used for Assignment of Value	Interpretation
Immunohistochemistry (IHC)		
0	No cells with a positive reaction were detected in the visual field	-
+	Few cells with a positive reaction were detected in the visual field	-
++	Moderate number of cells with a positive reaction were detected in the visual field	-
+++	Numerous cells with a positive reaction were detected in the visual field	-
++++	Abundant cells with a positive reaction were detected in the visual field	-
Chromogenic In-Situ Hybridization (CISH)		
0	1 to 5 green signals (copies) per nucleus detected in the cells	No amplification
+	5 to 6 green signals (copies) per nucleus detected in the cells	Low-level amplification
++	6 to 10 green signals (copies) per nucleus detected in the cells	Moderate-level amplification
+++	>10 green signals (copies) detected in the cells	High-level amplification
++++	Large cluster of green signals (copies) per nucleus detected in the cells	High-level amplification

**Table 3:** Results by patient (semi-quantitative grading scale) for the IHC staining.

Patient Number	Epithelium			Connective Tissue			Endothelium		
	*bFGF*	*FGFR1*	*FOXE1*	*bFGF*	*FGFR1*	*FOXE1*	*bFGF*	*FGFR1*	*FOXE1*
1	+	++	+	+	0	++++	+	0	++++
2	+	+	+	+	+	++++	+	0	++++
3	0	0	0	0	+++	+	0	0	++
4	+	+	++	+	+	++++	+	0	++++
5	++	++	+	+	+	++++	++	0	++++
6	+	++	+++	+	+	+++	++	0	++++
7	++	++	+++	0	0	++	+	0	+++
8	++	++	+++	0	+++	+++	+	0	++++
9	+	++	+	+	+	+++	+	0	++++
10	+	+	0	+	0	+++	++	0	++++
11	++	++	+++	++	+	+++	++	0	++++
12	0	0	0	0	0	+++	+	0	++++
Mean	1.17	1.42	1.50	0.75	1.00	3.08	1.25	0.00	3.75
SD *	0.72	0.79	1.24	0.62	0.45	0.90	0.62	0.00	0.62
CV% **	62.0	56.0	83.0	83.0	45.0	29.0	50.0	0.00	17.0

* SD—standard deviation; ** CV%—coefficient of variation (rounded off in percent) calculated as the ratio of the standard deviation and mean. Note that the mean was calculated in terms of the number of pluses, i.e., in semi-quantitative terms.

**Table 4:** Results by patient (semi-quantitative grading scale) for the CISH analysis.

Patient Number	Epithelium			Connective Tissue			Endothelium		
	*FGFR1*	*FGFR2*	*FOXO1*	*FGFR1*	*FGFR2*	*FOXO1*	*FGFR1*	*FGFR2*	*FOXO1*
1	0	0	0	0	0	0	0	0	0
2	++	++	0	++	+	0	0	0	0
3	0	+	0	0	0	0	0	+	0
4	0	0	0	0	0	0	0	0	0
5	++	++	+	+	+	0	0	0	0
6	0	0	0	0	0	0	+	0	0
7	+	+	+	0	0	0	0	0	0
8	+	0	0	++	+	0	0	0	0
9	+	+	0	+	+	0	0	0	0
10	+	0	+	+	0	0	+	0	0
11	+	++	0	+	+	0	0	0	0
12	0	0	0	0	0	0	0	0	0
Mean	0.75	0.75	0.25	0.67	0.42	0.00	0.17	0.08	0.00
SD *	0.74	0.87	0.45	0.78	0.51	0.00	0.39	0.28	0.00
CV% **	99.0	116.0	180.0	116.0	121.0	0.00	229.0	350.0	0.00

* SD—standard deviation; ** CV%—coefficient of variation (rounded off in percent) calculated as the ratio of the standard deviation and mean. Note that the mean was calculated in terms of the number of pluses, i.e., in semi-quantitative terms.

## Data Availability

All datasets used/analyzed in the present study are presented in the results sections of the article.
